# Immunoblot-based activity assay for heme-containing histidine kinases

**DOI:** 10.1007/s00775-026-02145-0

**Published:** 2026-04-29

**Authors:** Grant W. Larson, Eaindra Yee, Ambika Bhagi-Damodaran, Anoop Rama Damodaran

**Affiliations:** Department of Chemistry, University of Minnesota, Twin Cities, Minneapolis, MN-55455 USA

**Keywords:** Heme, Histidine kinase, Autophosphorylation, Redox signaling, Tuberculosis

## Abstract

Histidine kinases (HKs) are essential bacterial signal transduction proteins and attractive drug targets due to their critical cellular functions. Direct measurement of their autophosphorylation activity is crucial for developing inhibitors and advancing disease treatments. While [γ-^32^P]-ATP radiolabeling has long been a conventional method for kinase activity measurements, its reliance on ^32^P introduces inherent limitations. The isotope’s short half-life imposes time-sensitive constraints on experiments, and stringent radiation safety and compliance requirements significantly increase operational costs. Several alternative methods including fluorescent phosphate-binding probes and fluorescence- or luminescence-based antibodies have been developed for HKs that overcome these challenges. However, for heme-based HKs, these alternatives are prone to interference from the luminescence of heme or fluorescence from a minor fraction of mismetalated Zn^II^-bound protoporphyrin IX that is retained on the protein after gel electrophoresis. In this work, we address this interference by employing a near-IR (NIR) fluorophore-labeled secondary antibody and combining it with an ATPγS-utilizing immunoblot-based method to demonstrate ligation dependent thiophosphorylation activities of two heme-based HKs, DosS and GcHK. Consistent with previous studies, our NIR fluorescence-based immunoblot method reveals that GcHK is activated by ligand binding (O_2_/CO), whereas the O_2_-sensor DosS is inhibited upon binding O_2_. Overall, this easy-to-implement method simplifies and democratizes activity measurements of heme-based HKs.

**Supplementary Information:**

The online version contains supplementary material available at 10.1007/s00775-026-02145-0.

## Introduction

Histidine kinases (HKs) are ubiquitous signal transduction proteins found in bacteria, fungi, and plants. They play a crucial role in cellular adaptation by sensing and responding to changes in their microenvironment [1–3]. These proteins typically consist of a sensor domain, a linker domain, a dimerization and histidine phosphotransfer (DHp) domain, and a catalytic/ATP-binding (CA) domain. Upon detecting a stimulus, the sensor domain triggers conformational changes that propagate through the linker and DHp domains, which in turn promotes autophosphorylation at a conserved histidine residue within the DHp domain [4]. The phosphoryl group is then transferred to a response regulator which modulates gene transcription by directly binding to the organism’s DNA (Fig. S1) [5]. Given the essential role of HKs in bacterial signaling and adaptation, they represent promising therapeutic targets for antibiotic discovery [6]. Developing HK inhibitors requires reliable, accessible, and scalable autophosphorylation assays. Radiometric assays that employ [γ-^32^P]-ATP have been generally considered as the gold standard for autophosphorylation assays (Fig. S2) [7, 8]. However, the short half-life of ^32^P coupled with radiation safety and compliance challenges along with high reagent and operational costs have motivated the development of alternative approaches. In turn, approaches including fluorescence- or luminescence-based phosphohistidine antibodies [9], and fluorescent [10] or electrophoretic mobility shift [11–13] based phosphate-binding probes have been developed.

Despite the development of these alternative approaches, heme-based HKs have predominantly been investigated using the radiometric approach [14–22]. A select few studies have employed electrophoretic mobility-based assays that utilize gels loaded with phosphate-binding molecules (Fig. S3) [11, 13]. Luminescence or fluorescence-based kinase assays have not been employed for heme-based HKs, likely due to interference from the luminescence of heme or background fluorescence from a minor fraction of mismetalated Zn^II^-bound protoporphyrin IX (Zn-PPIX) that are retained on the protein even after gel electrophoresis. We note that heme proteins recombinantly expressed in *E. coli* may contain a minor subpopulation bound to Zn-PPIX rather than the functional Fe-PPIX cofactor due to the limited bioavailability of Fe^2+^ in aerobic cultures, increased iron demand during the induction phase of desired heme protein, and the promiscuity of ferrochetalase to Zn^2+^ incorporation [23–28]. Even a minor fraction of Zn-PPIX retained in the protein can introduce significant background fluorescence interference under standard visible-light fluorescent detection settings [29]. Furthermore, the low stability of phosphorylated histidine precludes the use of thermal denaturation or acidification protocols for complete removal of Zn-PPIX or heme [30].

In this work, we demonstrate that fluorescent detection of kinase activity in heme-based HKs is achievable by employing a near-IR (NIR) fluorophore-labeled secondary antibody, which circumvents any interference from Zn-PPIX related fluorescence. Combining NIR fluorescent detection with a previously developed ATPγS-based immunoblotting method [31, 32] for detecting thiophosphohistidine (tpHis, a phosphohistidine analog with significantly enhanced stability [30]), we provide a robust fluorescence-based kinase activity assay that is reliable and easy to implement. We apply this method to characterize the ligation-state dependent thiophosphorylation activities of two prototypical heme-based HKs, *Mycobacterium tuberculosis* DosS [21, 33–35] and *Anaeromyxobacter sp. Fw 109-5* GcHK [36, 37]. Consistent with previous studies, our immunoblot-based activity assays show that GcHK’s autothiophosphorylation activity is significantly enhanced when its heme cofactor is bound to O_2_ and CO, as compared to its unligated ferrous state [36, 38]. Additionally, DosS’s autothiophosphorylation activity is significantly enhanced when its heme cofactor is in the unligated ferrous state or bound to CO, compared to its O_2_-bound state [14].

## Methods

### DosS expression

While the precise subcellular localization of DosS remains to be definitely established [15, 39], several research groups [14, 20, 40, 41] have recombinantly expressed and purified it as a soluble protein from *E.coli* without any detergent or amphipols and have verified that the purified protein retains ligation-state dependent kinase activity. Based on these studies, we recombinantly expressed DosS in *E coli *cells and purified it without any detergent or amphipols. Briefly, BL21(DE3) *E. coli* cells (ThermoFisher Scientific) were transformed with a pET23a(+) plasmid containing a gene for *Mycobacterium tuberculosis* DosS (Accession: P9WGK3) with an N-terminal 6xHis tag and a TEV protease site (GenScript) and a pACYC-GroEL/ES-TF plasmid containing the chaperonin GroEL/ES (Addgene 83923). Transformed cells were plated on LB agar plates containing 100 µg/mL ampicillin and 37 µg/mL chloramphenicol. Primary cultures (100 mL 2XYT media containing 100 µg/mL ampicillin and 37 µg/mL chloramphenicol) were inoculated with a single colony from an agar plate. They were grown overnight at 37 °C while shaking at 220 rpm. Secondary cultures (1 L 2XYT media containing 100 µg/mL ampicillin and 37 µg/mL chloramphenicol) were inoculated with 25 mL of primary culture and were grown at 37 °C while shaking at 220 rpm. The cell density was monitored until the OD_600_ reached 0.5–0.7. Afterwards, the shaker conditions were changed to 30 °C and 170 rpm. Heme precursor molecules, 5-aminolevulinic acid and ferrous ammonium sulfate, were added to the cultures at 0.5 mM and 0.1 mM, respectively. Protein expression was induced with 0.5 mM isopropyl-1-thio-β-D-galactopyranoside (IPTG, GoldBio) and was allowed to proceed for 18 h. Cells were harvested via centrifugation at 2000 rpm for 30 min at 4 °C (Beckman Coulter). Overall, our DosS expression and purification methods were similar to those reported in previous work [42, 43].

### HK853 expression

BL21(DE3) *E. coli* cells were transformed with a pET28a(+) plasmid (generously provided by the Carlson lab at the University of Minnesota) containing a gene for the cytosolic portion of HK853 (Accession: Q9WZV7; amino acids 234–489) with an N-terminal 6xHis tag and a thrombin protease site (GenScript). Transformed cells were plated on LB agar plates containing 50 µg/mL kanamycin. Primary cultures (100 mL 2XYT media containing 50 µg/mL kanamycin) were inoculated with a single colony from an agar plate. They were grown overnight at 37 °C while shaking at 220 rpm. Secondary cultures (1 L 2XYT media containing 50 µg/mL kanamycin) were inoculated with 25 mL of primary culture and were grown at 37 °C while shaking at 220 rpm. The cell density was monitored until the OD_600_ reached 0.5–0.7. Afterwards, the shaker conditions were changed to 18 °C and 170 rpm. Protein expression was induced with 0.5 mM IPTG and was allowed to proceed for 18 h. Cells were harvested via centrifugation at 2000 rpm for 30 min at 4 °C (Beckman Coulter).

### GcHK expression

BL21(DE3) *E. coli* cells were transformed with a pET28a(+) plasmid containing a gene for *Anaeromyxobacter sp. Fw 109-5* GcHK (Accession: A7HD43) with an C-terminal 6xHis tag (GenScript). Transformed cells were plated on LB agar plates containing 50 µg/mL kanamycin. Primary cultures (100 mL 2XYT media containing 50 µg/mL kanamycin) were inoculated with a single colony from an agar plate. They were grown overnight at 37 °C while shaking at 220 rpm. Secondary cultures (1 L 2XYT media containing 50 µg/mL kanamycin) were inoculated with 25 mL of primary culture and were grown at 37 °C while shaking at 220 rpm. The cell density was monitored until the OD_600_ reached 0.5–0.7. Afterwards, the shaker conditions were changed to 18 °C and 170 rpm. Protein expression was induced with 0.5 mM IPTG and was allowed to proceed for 18 h. Cells were harvested via centrifugation at 2000 rpm for 30 min at 4 °C (Beckman Coulter).

### DosS, HK853, and GcHK purification

Cell pellets containing either DosS, GcHK or HK853 were resuspended in 50 mM Tris (pH 7.5), 250 mM sodium chloride, and 1% Triton X-100 and lysed via sonification (Fisher Scientific) while on ice. The crude lysate was centrifuged for 1 h at 20,000 rpm and 4 °C (Beckman Coulter). Hemin (30 µM) was added to just the GcHK lysate to prepare the heme-bound protein species. The supernatant was filtered using a 0.44 μm syringe filter (Sartorius) and applied to a 5 mL HisTrap FF column (GE Healthcare) at a rate of 2 mL/min. The column was washed with 75 mL 50 mM Tris (pH 7.5), 250 mM sodium chloride, and 20 mM imidazole at a rate of 2 mL/min. Recombinant protein was eluted with 50 mM Tris (pH 7.5), 250 mM sodium chloride, and 400 mM imidazole. The semi-pure DosS solution was oxidized with 2.5 mM potassium ferricyanide (III). Excess oxidant was removed by buffer exchanging the protein solution into 50 mM Tris pH 8.0, 100 mM NaCl, and 5% glycerol via gel filtration through a PD-10 column (Cytiva). Concentrated GcHK or DosS solutions were purified via size exclusion chromatography (SEC) with a HiPrep 16/60 Sephacryl S-200 HR column (GE Healthcare). HK853 was purified via SEC with a HiLoad 26/600 Superdex 75 pg column (GE Healthcare). Aliquots of the protein solution were flash-frozen in liquid nitrogen and stored at -80 °C.

### Autophosphorylation and autothiophosphorylation assays

Solutions of ferrous DosS were prepared anaerobically (Coy Lab Products) by reducing ferric DosS with sodium dithionite, and removing the excess dithionite and its oxidized byproducts using a PD-10 desalting column and 10 kDa centricon filters. Ferrous-CO DosS was prepared by adding 20 molar equivalents of CORM-A1 (Sigma Aldrich) to the ferrous protein. Ferrous-O_2_ DosS was prepared by exposing ferrous DosS to air for 5 min. All redox and ligation states of DosS were confirmed via UV-Vis (Agilent). Autophosphorylation reactions were performed in 50 mM Tris (pH 8.0), 50 mM potassium chloride, 10 mM magnesium (II) chloride, and 10% glycerol while shaking at 300 rpm at 20 °C. Reactions contained 5 µM HK853, DosS, or GcHK and were initiated by adding 1 mM adenosine triphosphate (ATP; Millipore Sigma) for autophosphorylation assays or adenosine 5′-[γ-thio]triphosphate (AGS; Cayman Laboratories) for autothiophosphorylation assays. At designated time points, aliquots of the reaction mixture were removed and quenched by adding 100 mM EDTA. Quenched autophosphorylation reaction mixtures were carried on to gel electrophoresis. The autothiophosphorylated proteins in the quenched reaction mixtures were modified with 3 mM para-nitrobenzyl mesylate (PNBM, Astatech). The modification reaction proceeded for 90 min, shaking at 300 rpm at 20 °C.

### PhosBind SDS-PAGE

A 10% Tris-glycine polyacrylamide gel with 50 µM PhosBind acrylamide (APExBIO) was prepared. Phosphorylated protein samples were applied to the gel and separated with Tris-glycine running buffer (pH 8.8). The gel was stained with Coomassie and imaged with an Odyssey M imager (LI-COR) using the 700 nm channel.

### Western blot of autophosphorylation samples

Proteins were separated via polyacrylamide gel electrophoresis using an 8% Tris-Glycine gel and Tris-Glycine running buffer (pH 8.8). The proteins were transferred to a nitrocellulose membrane, which was dried at 37 °C for 30 min to fix the proteins to the membrane.

### Immunoblotting autophosphorylation samples

Membranes from Western blotting were rehydrated with TBS and blocked with Intercept (TBS) protein-free blocking buffer (LI-COR) for 60 min at room temperature while rocking. Primary antibody solution was prepared by diluting rabbit anti-thiophosphoester antibody (Abcam, ab92570) in Intercept T20 (TBS) protein-free antibody diluent. The membranes were incubated with primary antibody solution for 16 h at 4 °C while rocking, washed four times with TBST (TBS + 0.1% Tween 20), and incubated with the secondary antibody solution containing goat anti-rabbit IgG Alexa Fluor Plus 800 (ThermoFisher Scientific, A32735), for 60 min at room temperature. The membranes were washed four times with TBST and dried at 37 °C for 60 min before imaging on an Odyssey M imager (LI-COR) using the 800 nm channel. The images were processed and analyzed using Empiria Studio 3.0 (LI-COR). Subsequently, the membranes were stained with SYPRO Ruby protein blot stain (ThermoFisher Scientific) to determine the amount of total protein. The stained membranes were then imaged with an Odyssey M imager using the 488 A channel. The images were processed and analyzed using Empiria Studio 3.0.

### Fluorescence emission spectroscopy studies

50 µM DosS was prepared in 50 mM Tris pH 8.0, 100 mM NaCl, and 5% glycerol. The fluorescence spectrum of DosS protein was taken using the TECAN Spark microplate reader with the following settings: excitation wavelength at 420 nm, emission scan from 520 nm to 820 nm with step size of 1 nm, the gain was held at a constant value of 110, and the Z-position was calculated and optimized from the sample well.

## Results and Discussion

**Fig. 1:**
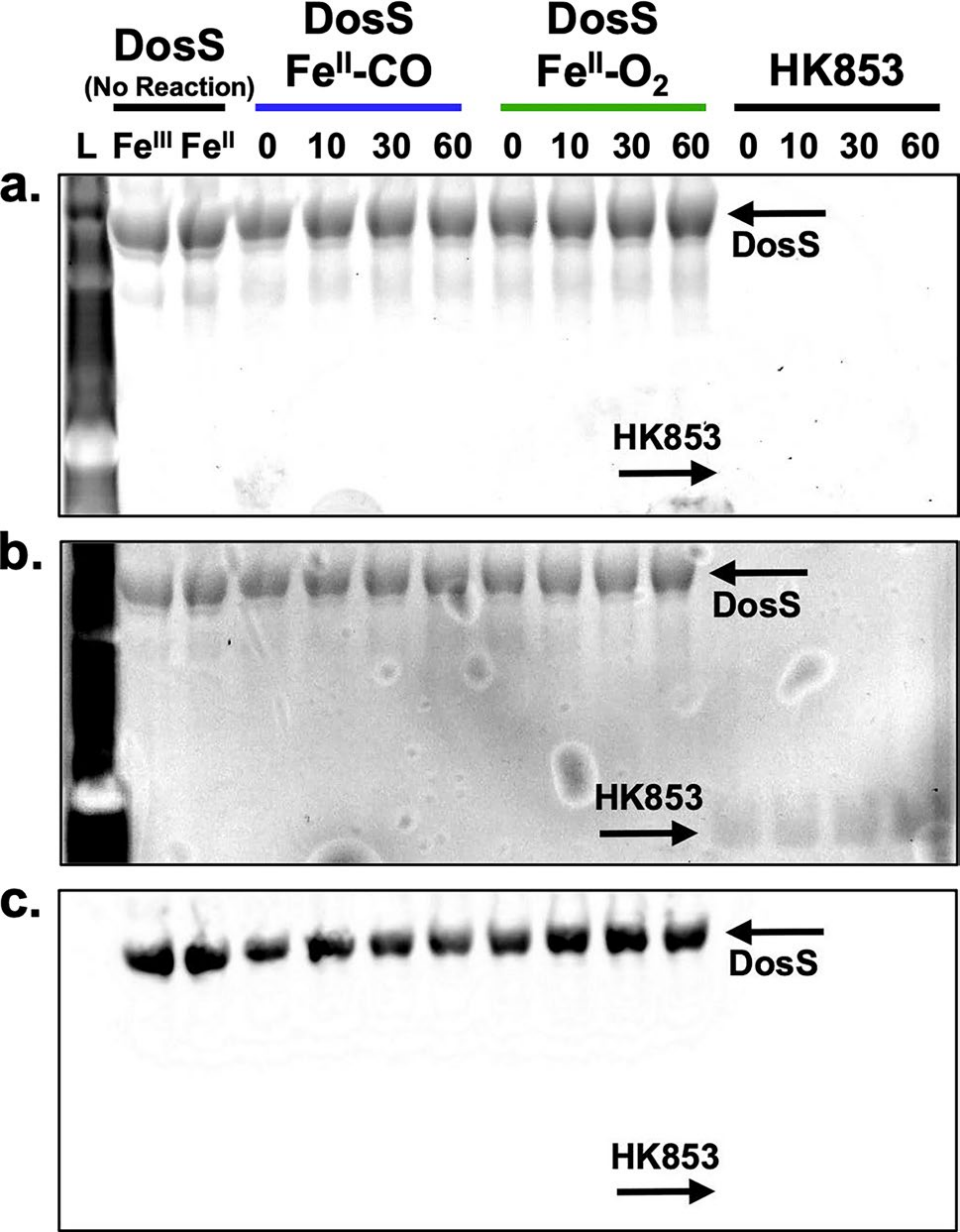
(**a**) Fluorescent visualization of an SDS-PAGE gel containing lanes loaded with unreacted ferric and ferrous DosS along with a time course of ferrous-CO, ferrous-O_2_ states of DosS and HK853 reacted with ATP (0, 10, 30 and 60 min). L indicates the lane for protein MW ladder. The fluorescence emission from the gel was measured with excitation at 532 nm and emission at 553 nm right after electrophoretic separation. (**b**) Gel in (a) was stained with PhosTag™ Aqua and imaged again with excitation at 532 nm and emission at 553 nm. (**c**) Luminescent visualization of proteins transferred to a membrane from a duplicate of the SDS-PAGE gel in (**a**) and reacted with luminol

To demonstrate the unsuitability of kinase assays that employ standard visible-light fluorescent or luminescent detection for heme-based HKs, we ran an SDS-PAGE gel with a set of samples that included unreacted ferric and ferrous DosS, and CO/O_2_-bound ferrous DosS reacted with ATP for up to 60 min (see Fig. S4 for UV-Vis spectra of each of these samples). We also included a heme-free HK construct ‒ a truncated form of *T. maritima* HK853 [44] that lacks its sensor domain and undergoes autophosphorylation even in the absence of a signal. Upon imaging the gel using visible-light fluorescent detection parameters prior to any fluorescent staining (Fig. 1a; see Coomassie stained gel in Fig. S6a), we observe a band corresponding to a strong and uniform fluorescence signal across all DosS samples (MW = 64 kDa). To understand the origin of this fluorescent signal, we measured the emission spectrum of DosS using a microplate reader. The spectrum reveals a primary peak at ~ 595 nm and a broad secondary peak centered at ~ 650 nm that are diagnostic markers for the presence of Zn-PPIX [29, 45] that can interfere with visible-light fluorescence (Fig. S5). On the other hand, HK853, which possesses no fluorescence under these imaging conditions, reveals clear lanes and the absence of any band at positions corresponding to its molecular weight of 32 kDa. Upon staining the gel with PhosTag™ Aqua stain (a phosphate-binding fluorophore) to assess autophosphorylation levels, we observe that background fluorescence obscures DosS signals (Fig. 1b, upper arrow). In contrast, HK853 reveals increasing auto-phosphorylation over the time course of the reaction (Fig. 1b, lower arrow). Next, we ran a duplicate SDS-PAGE gel, transferred it to a nitrocellulose membrane, and imaged the membrane with luminol using standard chemiluminescent detection without having carried out any immunoblotting with primary/secondary antibodies. We note that the DosS band is able to catalyze luminol oxidation, generating strong and unwanted luminescence (Fig. 1c, upper arrow; see SYPRO Ruby stained membrane in Fig. S6b). Again, HK853 reveals clear lanes and the absence of any luminescent bands at positions corresponding to its molecular weight (Fig. 1c, lower arrow). Taken together, these studies show that standard visible-light fluorescent and luminescent detection-based kinase assays are prone to interference when investigating heme-based HKs.

**Fig. 2:**
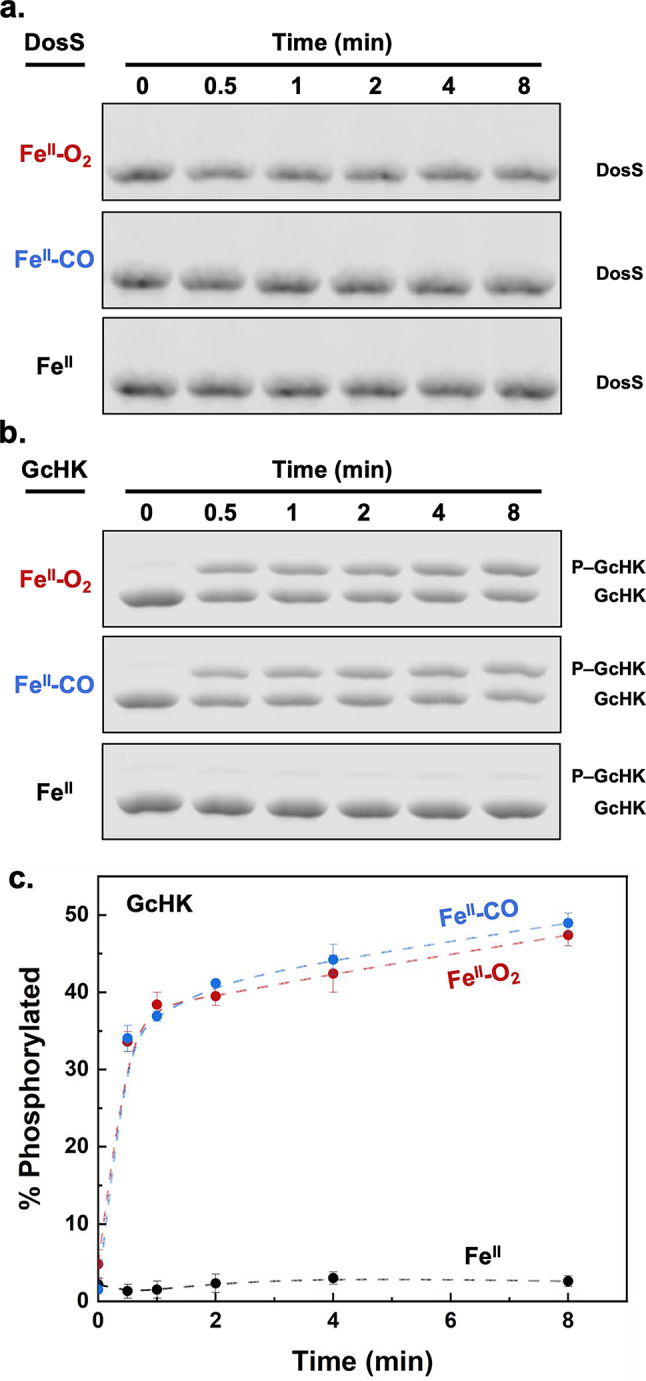
(**a**) Images of Coomassie-stained PhosBind polyacrylamide gels loaded with DosS in the Fe^II^-O_2_ (top), Fe^II^-CO (middle), and Fe^II^ (bottom) states. (**b**) Images of Coomassie-stained PhosBind polyacrylamide gels loaded with GcHK in the Fe^II^-O_2_ (top), Fe^II^-CO (middle), and Fe^II^ (bottom) states. Autophosphorylated GcHK is separated from the unphosphorylated protein forming two bands in different ratios. These bands are denoted as P–GcHK for phosphorylated GcHK or GcHK for non-phosphorylated GcHK. (**c**) A plot demonstrating the percentage of GcHK phosphorylated over time in different ligation states. The Fe^II^-CO (blue) and Fe^II^-O_2_ (red) states are active and reach approximately 50% phosphorylated after 8 min, while the Fe^II^ (black) state is inactive and does not demonstrate significant autophosphorylation. Dashed lines serve as guides to the eye

We next employed an electrophoretic mobility shift assay that incorporates a phosphate-binding molecule within SDS polyacrylamide gels to selectively slow the migration of phosphorylated proteins. This method resolves phosphorylated and non-phosphorylated proteins into distinct bands, enabling a ratiometric quantification of kinase activity [46]. More particularly, this assay has been applied to determine the kinase activity of heme proteins such as GcHK and FixL [12, 13]. We therefore applied this assay to investigate the O_2_-switched kinase activity of DosS. Specifically, DosS is an active kinase when its heme-based sensor is in the Fe^II^ or Fe^II^-CO states but is inhibited in the Fe^II^-O_2_ state. We performed autophosphorylation assays with 5 µM DosS in its Fe^II^, Fe^II^-CO, and Fe^II^-O_2_ states in 50 mM Tris (pH 8.0) buffer containing 1 mM ATP and 10 mM MgCl_2_. Aliquots from these assays were quenched with EDTA at specific time points and separated on a 10% Tris-glycine gel containing 50 µM PhosBind acrylamide. The gel was stained with Coomassie and imaged with an Odyssey M imager (LI-COR) using the 700 nm channel. However, none of the DosS samples, whether in the Fe^II^, Fe^II^-CO or Fe^II^-O_2_ states showed any separation into distinct phosphorylated and non-phosphorylated bands (Fig. 2a). As a positive control, we simultaneously performed the same assay with GcHK, a heme-based kinase for which this method has been previously demonstrated to work. In contrast to DosS, the GcHK samples are resolved into distinct bands (Fig. 2b), allowing for ratiometric quantification of their autophosphorylation activity (Fig. 2c). Consistent with previous reports, these results confirm that GcHK is active in the Fe^II^-O_2_ and Fe^II^-CO states but inactive in the Fe^II^ state. These studies suggest that the application of the phosphorylation-dependent mobility shift assay is not universal and requires protein-specific optimization.

**Fig. 3:**
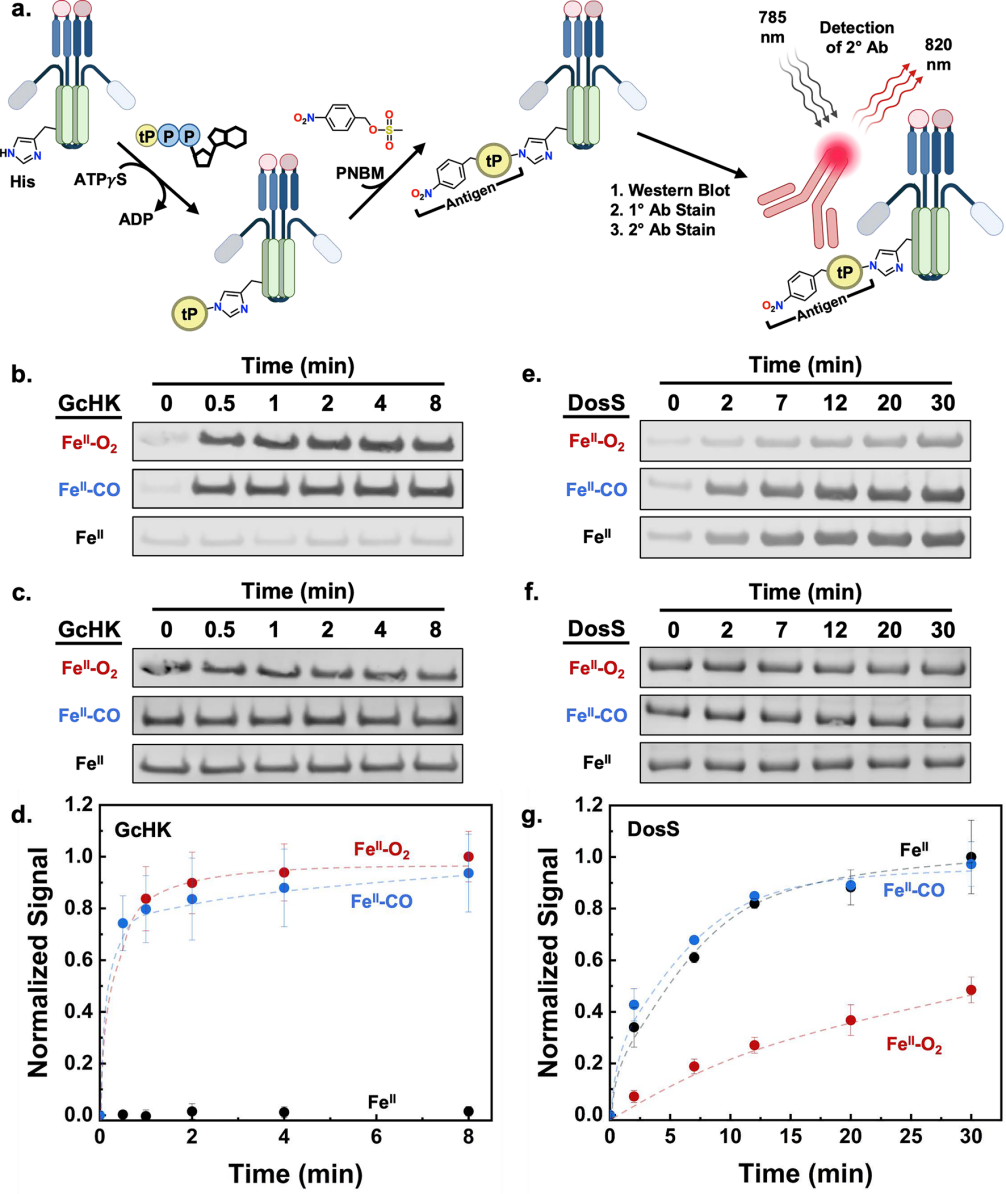
(**a**) Schematic depicting the use of NIR fluorescent secondary antibodies for immunoblot-based thiophosphorylation assays of heme-based HKs. The HK reacts with ATPγS, which transfers its thiophosphate (tP) group to the histidine residue of HK. The tP is chemically modified with PNBM to form the PNB-tP antigen. This modified protein sample is western blotted onto a nitrocellulose membrane and stained with primary and secondary antibodies. Our method makes use of a secondary antibody conjugated to a NIR labelled fluorophore, allowing for detection without interference from background fluorescence. Only one antibody is shown for clarity. Created with Biorender. (**b**) Fluorescent images of representative portions of an immunostained membrane containing Fe^II^-O_2_ (top), Fe^II^-CO (middle), and Fe^II^ (bottom) GcHK. Increasing band intensity from left to right indicates an increase in thiophosphorylation over time. The fluorescence was measured with excitation at 785 nm and emission at 820 nm. (**c**) Images of the membranes from (b) stained with SYPRO Ruby for total protein quantification. Even band density shows equivalent protein loading and transfer. The fluorescence was measured with excitation at 488 nm and emission at 590 nm. (**d**) Signal from the thiophosphorylation of Fe^II^-O_2_ (red), Fe^II^-CO (blue), and Fe^II^ (black) GcHK plotted with respect to time. Fe^II^-O_2_ and Fe^II^-CO GcHK exhibit enhanced thiophosphorylation compared to Fe^II^ GcHK. Dashed lines serve as guides to the eye. (**e**) Fluorescent images of representative portions of an immunostained membrane containing Fe^II^-O_2_ (top), Fe^II^-CO (middle), and Fe^II^ (bottom) DosS. Increasing band intensity from left to right indicates an increase in thiophosphorylation over time. The fluorescence was measured with excitation at 785 nm and emission at 820 nm. (**f**) Images of the membranes from (e) stained with SYPRO Ruby for total protein quantification. Even band density shows equivalent protein loading and transfer. The fluorescence was measured with excitation at 488 nm and emission at 590 nm. (**g**) Signal from the thiophosphorylation of Fe^II^-O_2_ (red), Fe^II^-CO (blue), and Fe^II^ (black) DosS plotted with respect to time. Fe^II^-CO and Fe^II^ DosS exhibit enhanced thiophosphorylation compared to Fe^II^-O_2_ DosS. Dashed lines serve as guides to the eye

The inability to resolve DosS phosphorylation with the mobility shift kinase assay demonstrated the need for an alternative approach to characterize its activity. NIR fluorescence-based immunoblots can circumvent challenges related to the background fluorescence in DosS samples. Accordingly, we combined NIR fluorescence detection with a previously developed immunoblot method that uses ATPγS instead of ATP [31, 32]. In this method, any tpHis formed in thiophosphorylation assays is converted to the biorthogonal epitope para-nitrobenzyl thiophosphate ester (PNB-tP), which is then recognized by a high-affinity anti-thiophosphate ester antibody [31] (Fig. 3a). The ATPγS-based immunoblot method has been utilized to detect and probe numerous kinases [31, 32], and offers several advantages over using phosphorylated-histidine detecting antibodies. First, the method is universal and applicable to histidine, serine, threonine, and tyrosine kinases. Second, focusing on HKs, tpHis is much more stable than phosphohistidine [30], thereby mitigating potential hydrolysis-related challenges during subsequent immunoblotting steps that could otherwise lead to unreliable activity measurements.

We began by investigating the time-dependent thiophosphorylation of GcHK in its Fe^II^, Fe^II^-CO, and Fe^II^-O_2_ forms. The thiophosphorylation reactions were quenched with EDTA at predetermined time points and were subsequently reacted with p-nitrobenzyl mesylate (PNBM). The protein samples were then subjected to SDS-PAGE for electrophoretic separation and Western blot transfer (see full gels in Fig. S7-S8). The band intensities of the Fe^II^-O_2_ and Fe^II^-CO GcHK increased over time (Fig. 3b, top and middle panels), indicative of auto-thiophosphorylation activity. Conversely, the Fe^II^ GcHK band intensities remained relatively the same (Fig. 3b, bottom panel), indicative of low auto-thiophosphorylation activity. Fluorescent signals were normalized to the Fe^II^-O_2_ 8 min sample and to the total amount of protein in each band, which was determined by staining the membrane with SYPRO Ruby (Fig. 3c). The GcHK activity trends observed in this NIR-based immunostaining method mirror those obtained using the mobility shift kinase assay, highlighting the suitability of this method for comparing the thiophosphorylation activity of a heme-based kinase in different ligation states.

Next, we employed this method to investigate the ligation-dependent thiophosphorylation activity of DosS over a 30-minute time course (Fig. 3e-f, see full gels in Fig. S9-S10). Lacking a 100% thiophosphorylated reference standard, the fluorescence intensity in our method cannot be directly converted to percentage thiophosphorylation. Instead, our method compares the time-dependent fluorescence signal intensity for different stimuli allowing us to determine the ratio of thiophosphorylation rates rather than absolute kinetics. After normalization to the total protein content, we observed that the fluorescent signals for Fe^II^ and Fe^II^-CO DosS increased at a rate four-fold greater than that of Fe^II^-O_2_ DosS (Fig. 3g). Overall, our studies showed that the Fe^II^ and Fe^II^-CO states are substantially more active at thiophosphorylation than the Fe^II^-O_2_ state, a trend consistent with previous radiolabeling-based kinase activity studies for DosS [14, 20, 21]. We also assessed the applicability of this assay in a dot-blot format and included the inactive H395Q DosS variant (Fig. S11-12). This mutant retains the heme-binding domain but lacks the target H395 histidine residue that undergoes thiophosphorylation in WT DosS and serves as a negative control. As expected, we observe no detectable thiophosphorylation in H395Q DosS across both ligation states (Fig. S12). This confirms that the signals measured in our auto-thiophosphorylation assays are due to functional thiophosphorylation events at the H395 position. Overall, these studies reveal the versatility of our NIR fluorescence-based thiophosphorylation assay to successfully capture both the ligand-induced activation of GcHK and the O_2_-specific inhibition of DosS. Furthermore, this work establishes the NIR-based thiophosphorylation assay as a robust, sensitive, and broadly applicable tool for conducting comparative studies of ligation-state dependent thiophosphorylation rates of heme-based kinases.

## Conclusion

Microbes utilize a diverse array of heme-based kinases to sense and signal changes in their redox environment [5]. Understanding how these kinases differentiate between various redox stimuli and transmit signals downstream to response regulators is a critical research focus from both biochemical and microbiological perspectives. Furthermore, the involvement of DosS-like HKs in infectious diseases makes them appealing drug targets [5]. This work introduces a NIR fluorescence-based thiophosphorylation assay to investigate the kinase activity of heme-based HKs in a low-cost, easy-to-implement, and scalable format. While the slower kinetics of thiophosphorylation [44] in this method may attenuate the magnitude of the stimuli-switched response compared to traditional phosphorylation assays, this study shows that the regulatory behavior for both DosS and GcHK remains intact. We believe that this assay paves the way for more rigorous characterization of heme-based HKs as well as the development of high-throughput modalities to discover their small-molecule inhibitors.

## Supplementary Information

Below is the link to the electronic supplementary material.

Supplementary Material 1

Supplementary Material 2

## Data Availability

Most of the data generated in this study is in form of blots/gels which are available as supporting info of this article.
