# Supplementary material for: Immunoblot-based activity assay for heme-containing histidine kinases
Source: J Biol Inorg Chem. 2026 Apr 29;31(3):163–71. doi: 10.1007/s00775-026-02145-0 (PMC13287260; doi:10.1007/s00775-026-02145-0)
Supplement: Supplementary file 1 — Supplementary Material 1 [file 775_2026_2145_MOESM1_ESM.pdf]

Supporting Information

Immunoblot-based activity assay for heme-containing histidine kinases

Grant W. Larson, Eaindra Yee, Ambika Bhagi-Damodaran*, Anoop Rama Damodaran*

Department of Chemistry, University of Minnesota, Twin Cities, Minneapolis, MN-55455, USA.

*Corresponding author emails: ambikab@umn.edu and rdanoop@umn.edu

Table of Contents

Supplemental Figures:

Supplemental Figure 1: Generic two-component system signal transduction pathway

Supplemental Figure 2: Structures of ATP and its analogs

Supplemental Figure 3: Structures of acrylamide and Phosbind™ acrylamide

Supplemental Figure 4: UV-Vis spectra of GcHK and DosS

Supplemental Figure 5: Fluorescence emission spectrum of DosS

Supplemental Figure 6: Total protein imaging of the gel and membrane in Figure 1

Supplemental Figure 7: GcHK immunostained membrane images from Figure 3

Supplemental Figure 8: GcHK SYPRO stained membrane images from Figure 3

Supplemental Figure 9: DosS immunostained membrane images from Figure 3

Supplemental Figure 10: DosS SYPRO stained membrane images from Figure 3

Supplemental Figure 11: Image of DosS dot blots

Supplemental Figure 12: Image of H395Q DosS dot blots

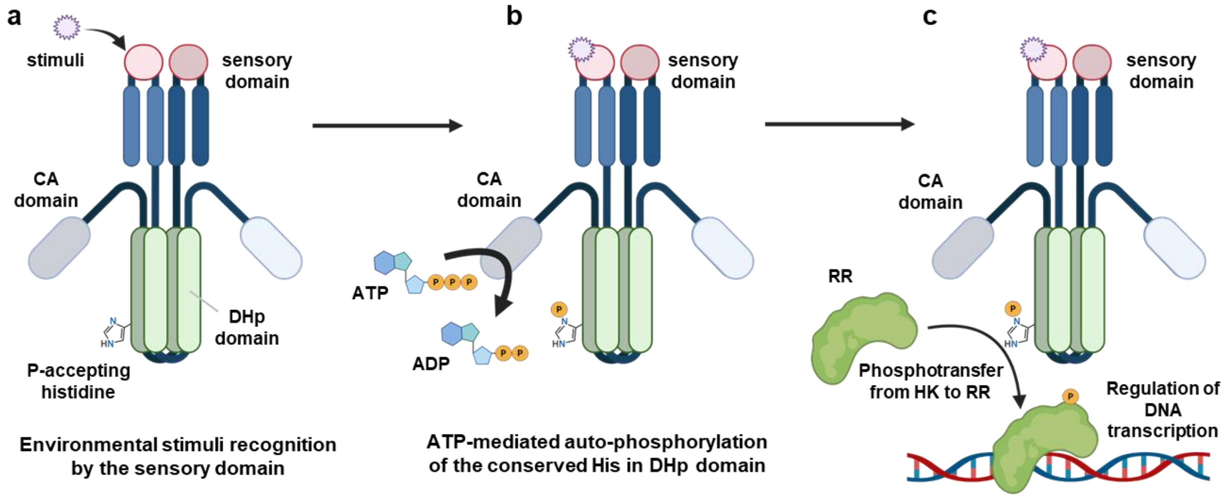

Supplemental Figure 1. A simplistic model of a Histidine Kinase (HK) and Response Regulator (RR) mediated bacterial TCS pathway. (**a**) An environmental stimulus is recognized by the HK's extracellular sensory domain. (**b**) Stimulus recognition by the sensory domain initiates ATP-mediated auto-phosphorylation of a conserved histidine in DHp domain. (**c**) The signal is further transmitted via phosphotransfer from HK to RR. The phosphorylated RR binds to the DNA and alters gene transcription to allow for cellular adaptation to the environmental stimuli. Note: This model demonstrates a cis auto-phosphorylation mechanism in panel (**b**) i.e. CA domain autophosphorylates DHp on the same HK monomer. The mechanism for DosS and GcHK autophosphorylation is not known. Created with Biorender.

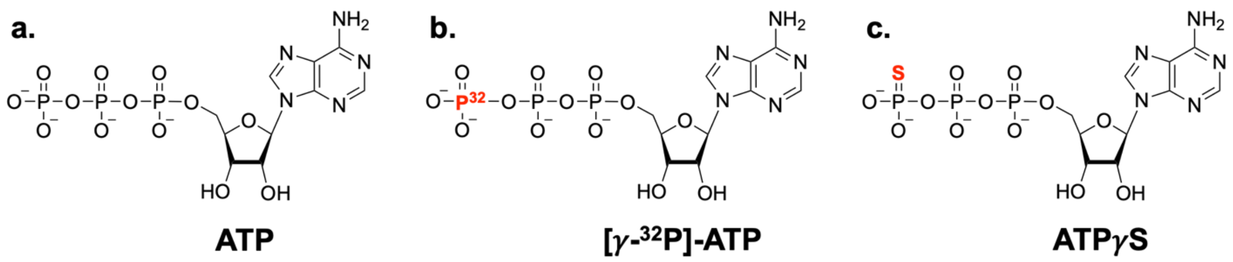

Supplemental Figure 2. Structures of (**a**) ATP, (**b**) [γ-^{32}P]-ATP, and (**c**) ATPγS. Structural differences in (**b**) and (**c**) are colored red.

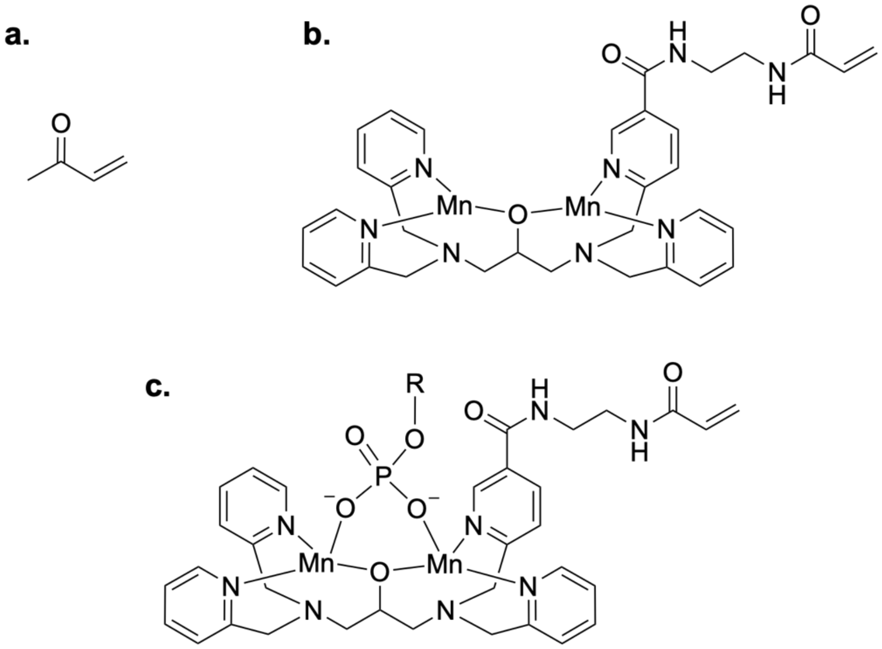

Supplemental Figure 3. Structures of (**a**) acrylamide, (**b**) PhosBind acrylamide, and (**c**) PhosBind acrylamide bound to phosphate.

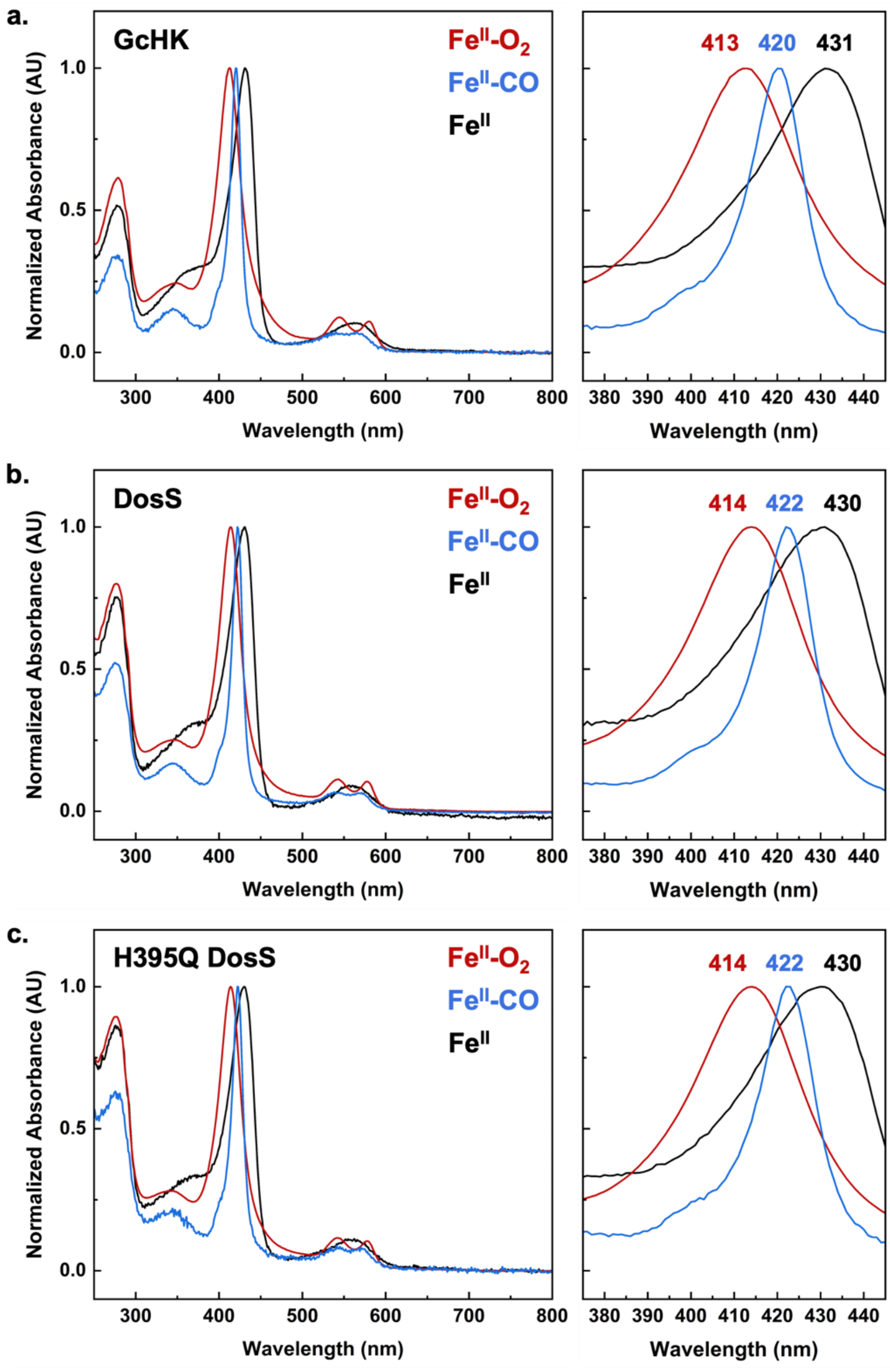

Supplemental Figure 4. UV-Vis spectra of (a) GcHK, (b) WT DosS, and (c) H395Q DosS in various ligation states. The left panels show the full UV-Vis spectra, while the right panel zooms into the heme Soret band of each species. The Soret band λ_{max} values of the GcHK samples are as follows: Fe^{II}-O_2 (413 nm), Fe^{II}-CO (420 nm), Fe^{II} (431 nm). The Soret band λ_{max} values of the WT and H395Q DosS samples are as follows: Fe^{II}-O_2 (414 nm), DosS Fe^{II}-CO (422 nm), DosS Fe^{II} (430 nm).

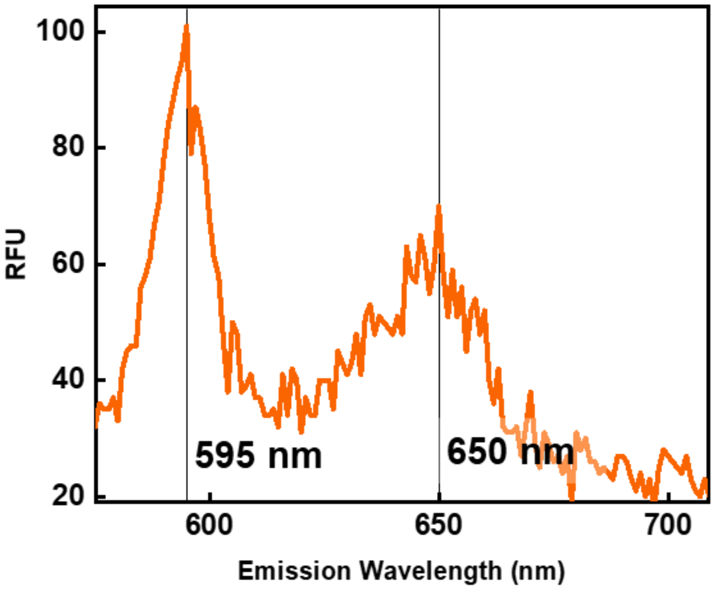

Supplemental Figure 5. Fluorescence emission spectrum of 50 μM WT DosS acquired with excitation at 420 nm.

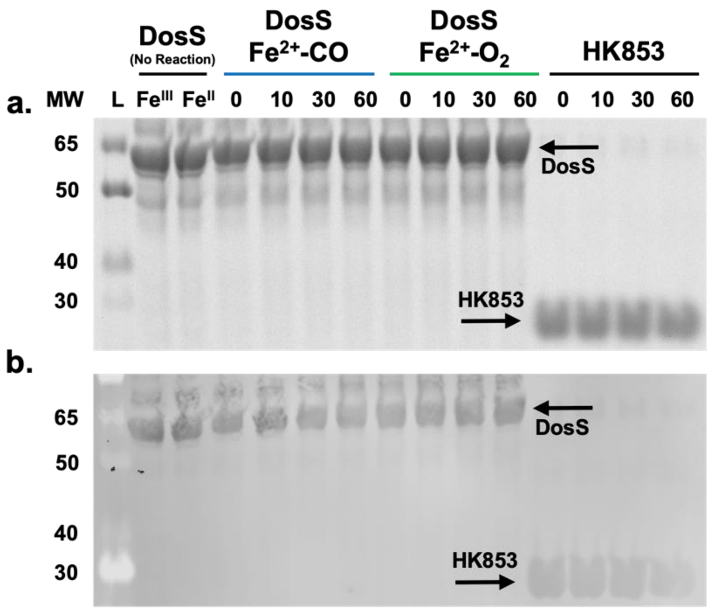

Supplemental Figure 6. (**a**) Images of gels from Figure 1a and 1b stained with coomassie to demonstrate the presence of HK853 and DosS in equivalent loading amounts. (**b**) Image of membrane from Figure 1c stained with SYPRO Ruby to demonstrate the presence of HK853 and DosS in equivalent loading amounts

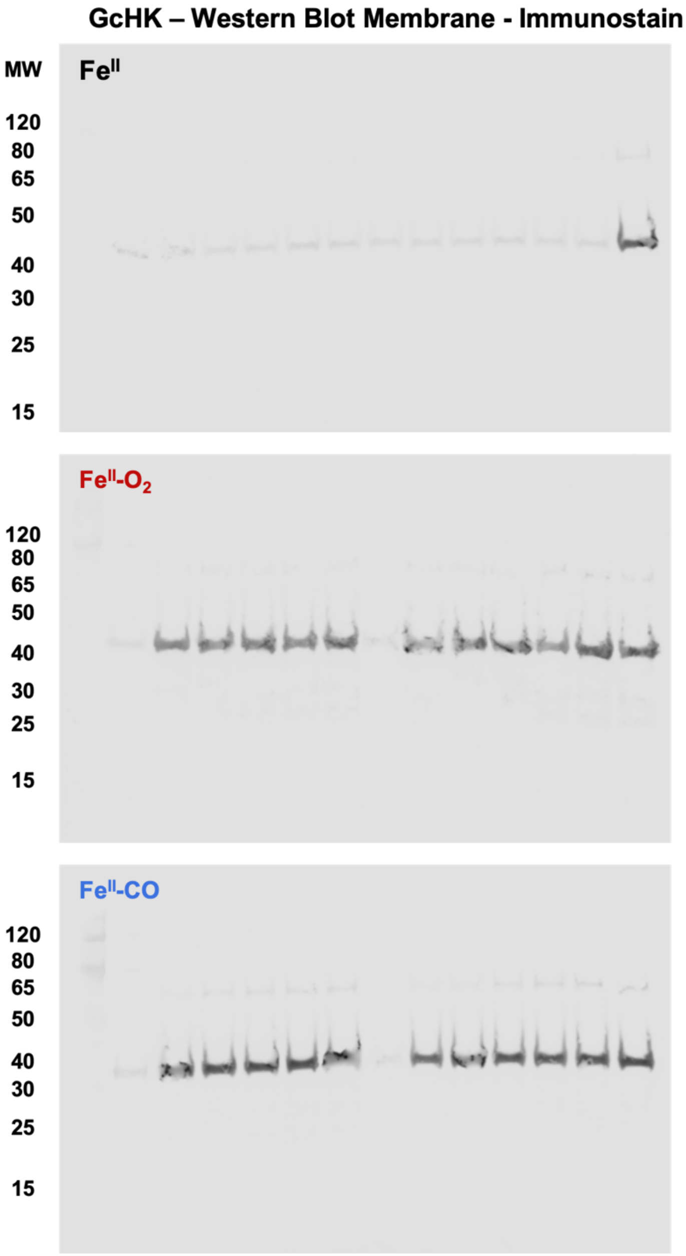

Supplemental Figure 7. Fluorescent image of a membrane with Fe^{II} (top), Fe^{II}-O_2 (middle), and Fe^{II}-CO (bottom) GcHK and immunostained with a rabbit monoclonal primary antibody specific to the PNBM and an anti-rabbit secondary antibody that is conjugated to a near-IR sensitive fluorophore. The fluorescence was measured with excitation at 785 nm and emission at 820 nm.

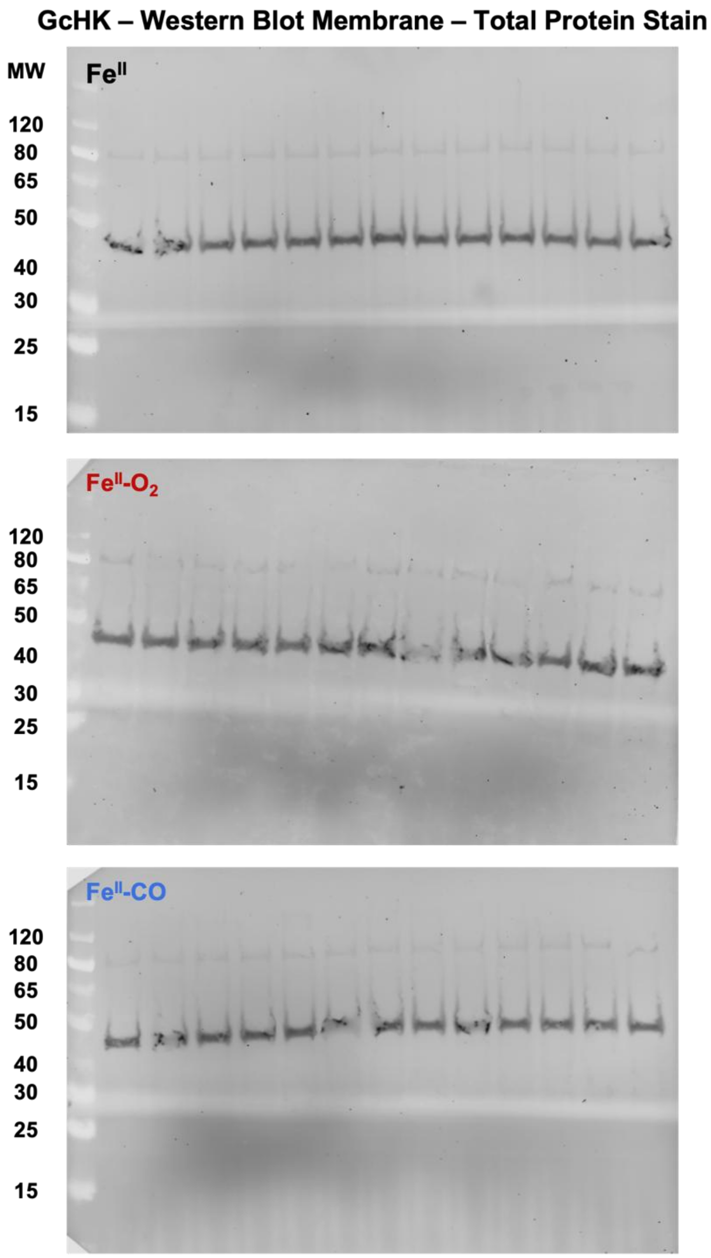

Supplemental Figure 8. Fluorescent image of the membranes in Fig. S7 stained with Sypro Ruby for total protein quantification. The fluorescence was measured with excitation at 488 nm and emission at 570 - 610 nm.

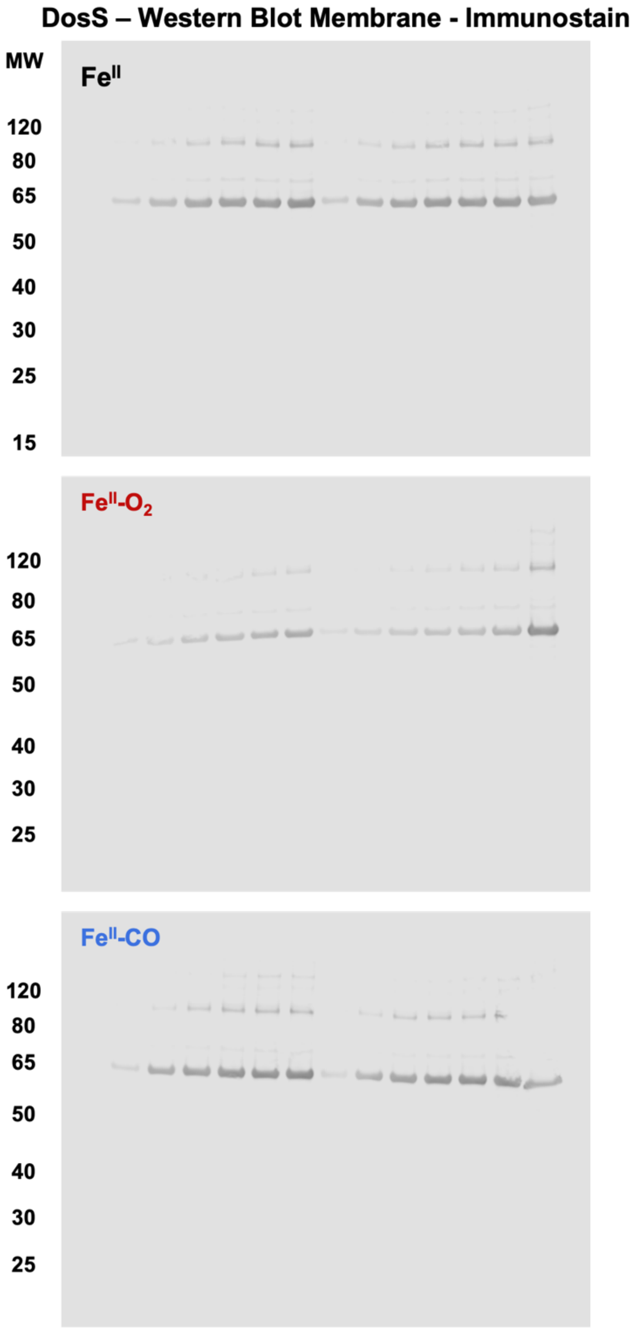

Supplemental Figure 9. Fluorescent image of a membrane with Fe^{II} (top), Fe^{II}-O_2 (middle), and Fe^{II}-CO (bottom) DosS and immunostained with a rabbit monoclonal primary antibody specific to PNBM and an anti-rabbit secondary antibody that is conjugated to a near-IR sensitive fluorophore. The fluorescence was measured with excitation at 785 nm and emission at 820 nm.

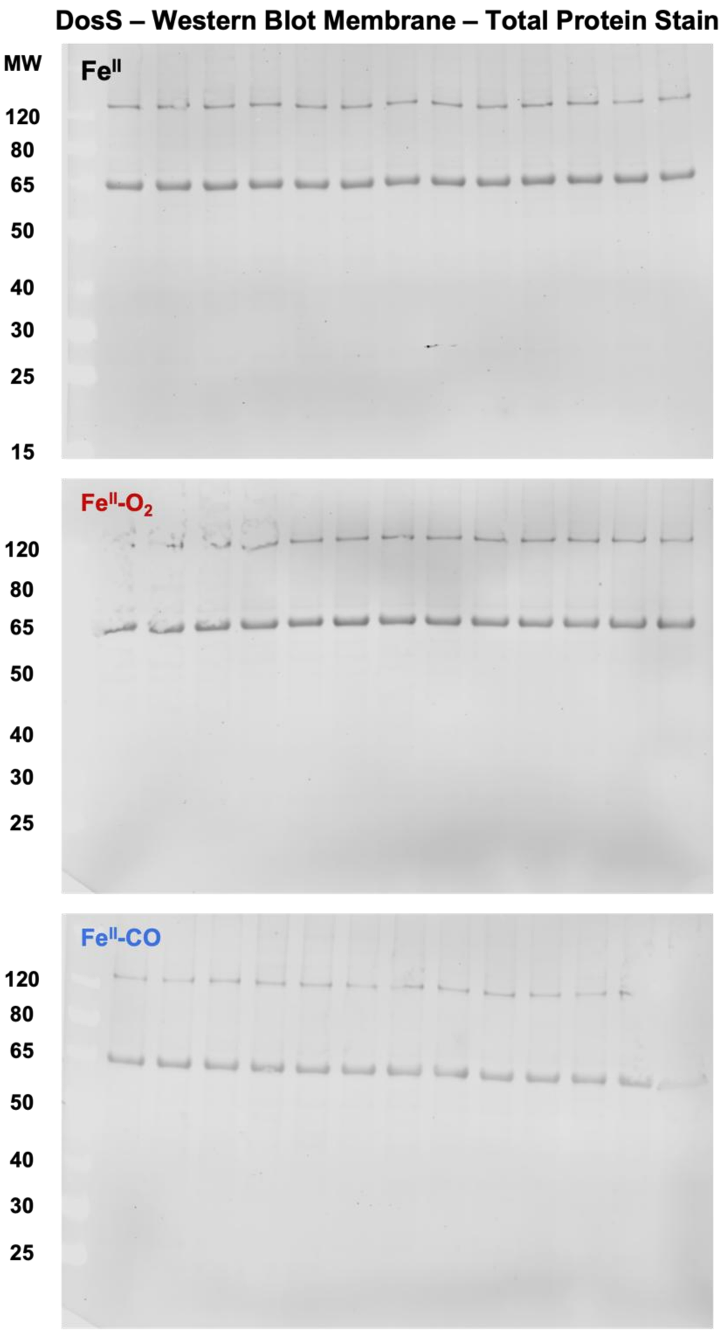

Supplemental Figure 10. Fluorescent image of the membranes in Fig. S9 stained with Sypro Ruby for total protein quantification. The fluorescence was measured with excitation at 488 nm and emission at 570 - 610 nm.

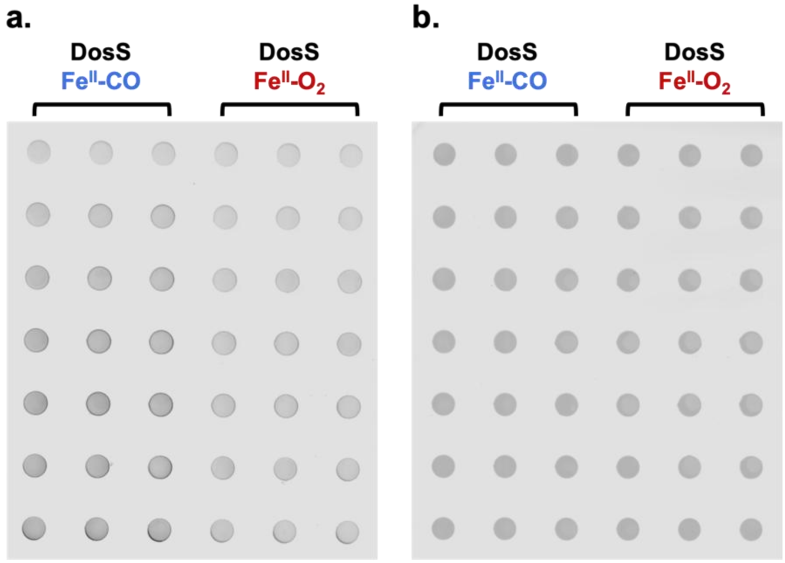

Supplemental Figure 11. (**a**) Fluorescent image of an immunostained dot blot membrane containing Fe^{II}-CO DosS (left) and Fe^{II}-O_2 DosS (right). Increasing dot intensity from top to bottom in the dots indicates an increase in thiophosphorylation over time. The fluorescence was measured with excitation at 785 nm and emission at 820 nm. (**b**) Image of the membranes from (a) stained with SYPRO Ruby for total protein quantification. Even dot density shows equivalent protein loading. The fluorescence was measured with excitation at 488 nm and emission at 590 nm.

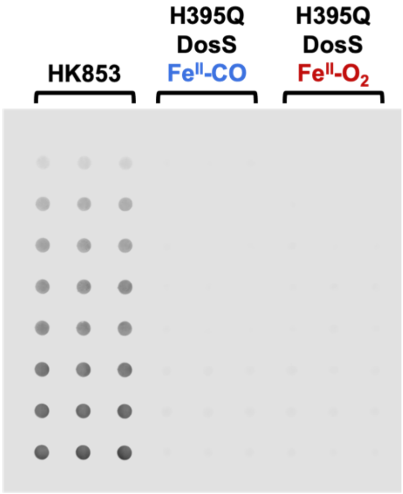

Supplemental Figure 12. Fluorescent image of an immunostained dot blot membrane containing HK853 (left), Fe^{II}-CO H395Q DosS (middle), and Fe^{II}-O_2 H395Q DosS (right). Increasing dot intensity from top to bottom in the HK853 dots indicates an increase in thiophosphorylation over time. The lack of fluorescence in the H395Q DosS samples is because this mutant lacks the histidine residue (H395) required for autothiophosphorylation. The fluorescence was measured with excitation at 785 nm and emission at 820 nm.
