# Supplementary material for: Immunoblot-based activity assay for heme-containing histidine kinases
Source: J Biol Inorg Chem. 2026 Apr 29;31(3):163–71. doi: 10.1007/s00775-026-02145-0 (PMC13287260; doi:10.1007/s00775-026-02145-0)

Supporting Information

Immunoblot-based activity assay for heme-containing histidine kinases

Grant W. Larson, Eaindra Yee, Ambika Bhagi-Damodaran*, Anoop Rama Damodaran*
Department of Chemistry, University of Minnesota, Twin Cities, Minneapolis, MN-55455, USA.
*Corresponding author emails: ambikab@umn.edu and rdanoop@umn.edu

This file contains the raw gel and dot-blot images used to generate the data in this manuscript

Fig. 1a

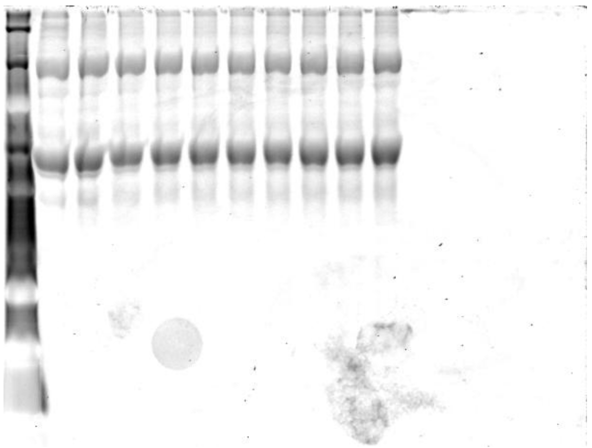

Fig. 1b

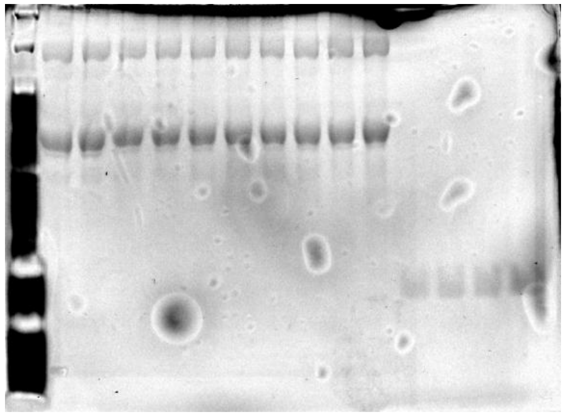

Fig. 1c

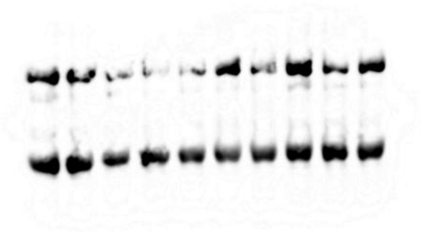

Fig. 2a

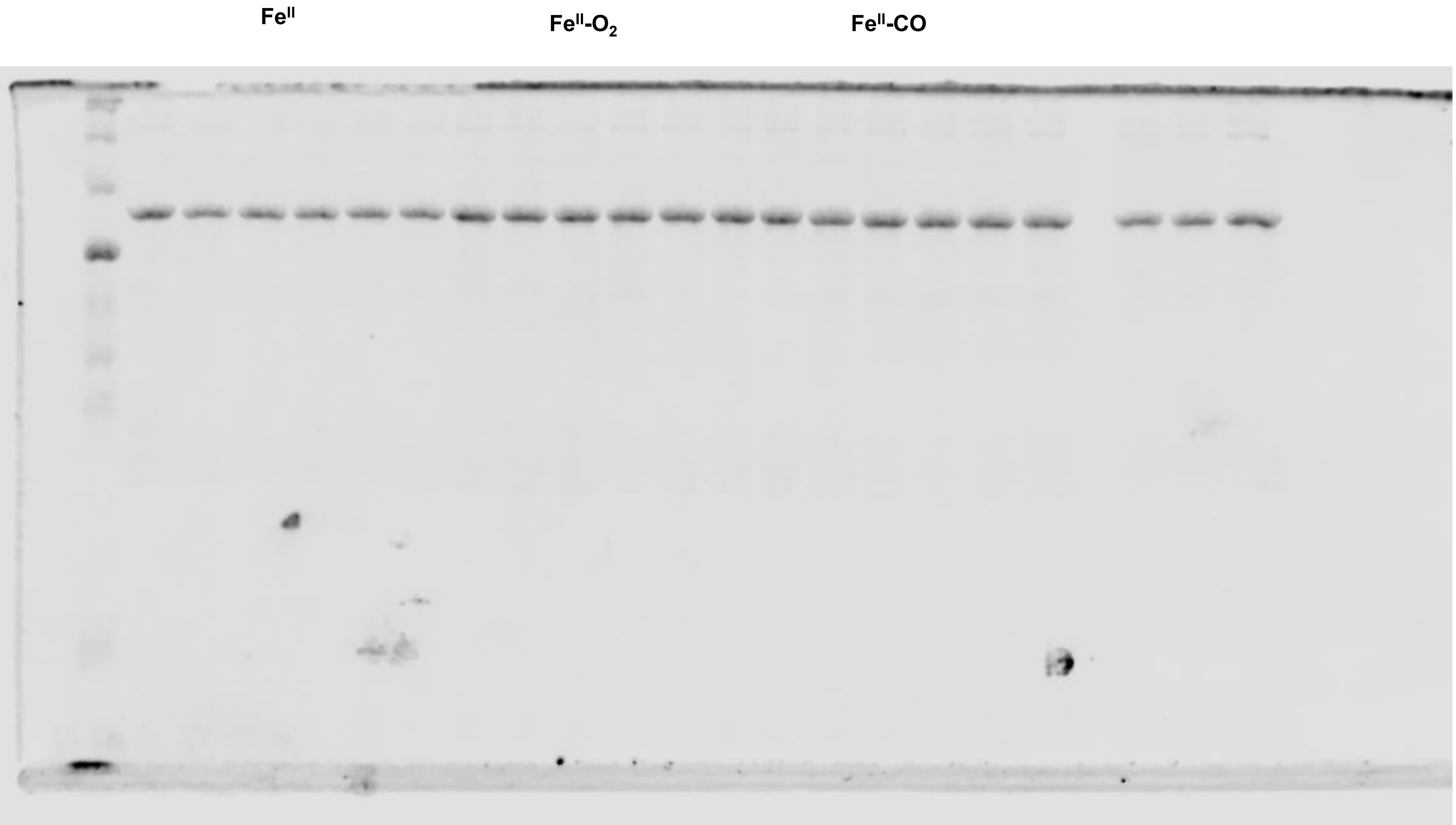

Fig. 2b

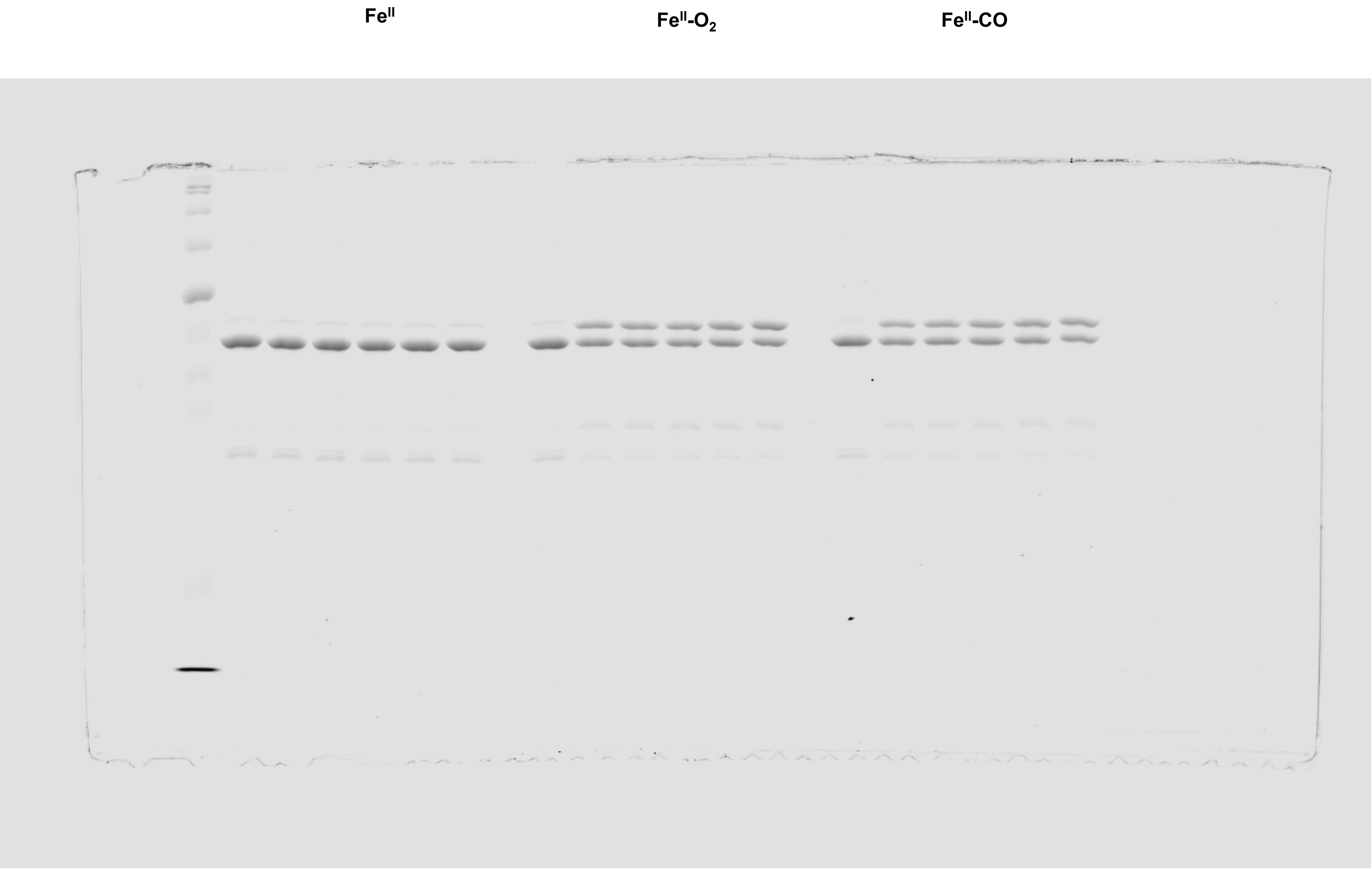

Fig. 3b

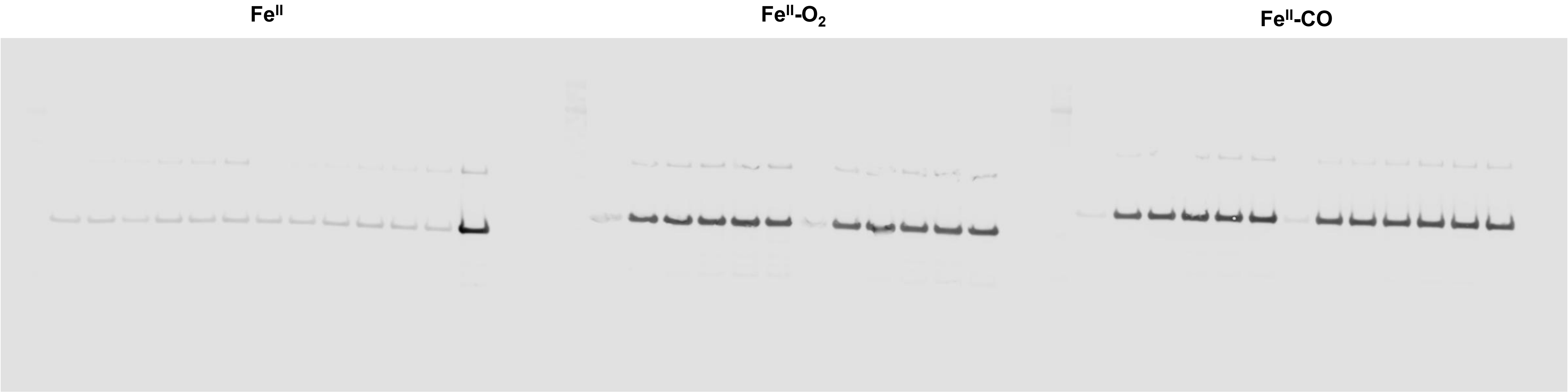

Fig. 3c

Fe^{II}-O_2

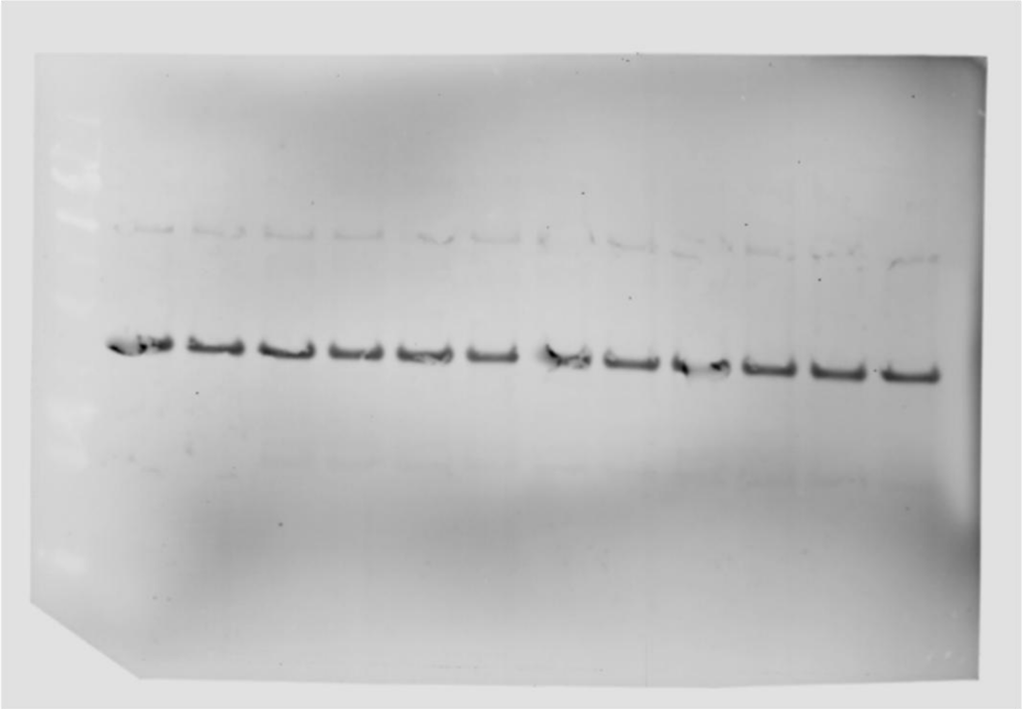

Fe^{II}

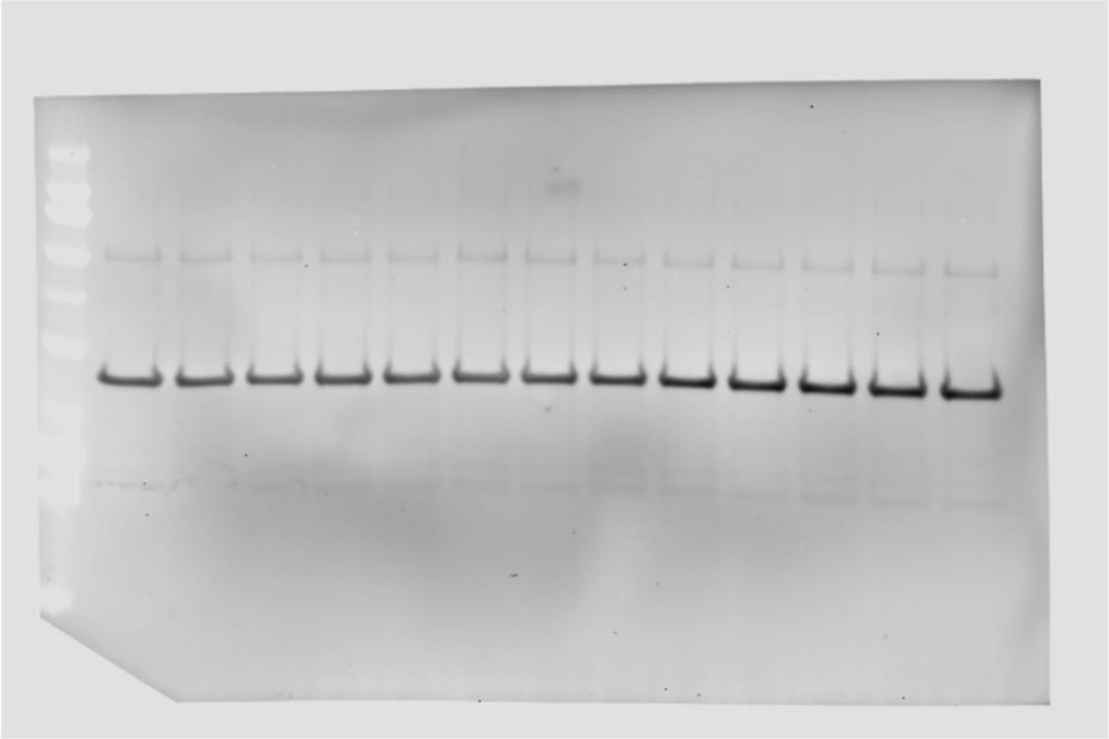

Fe^{II}-CO

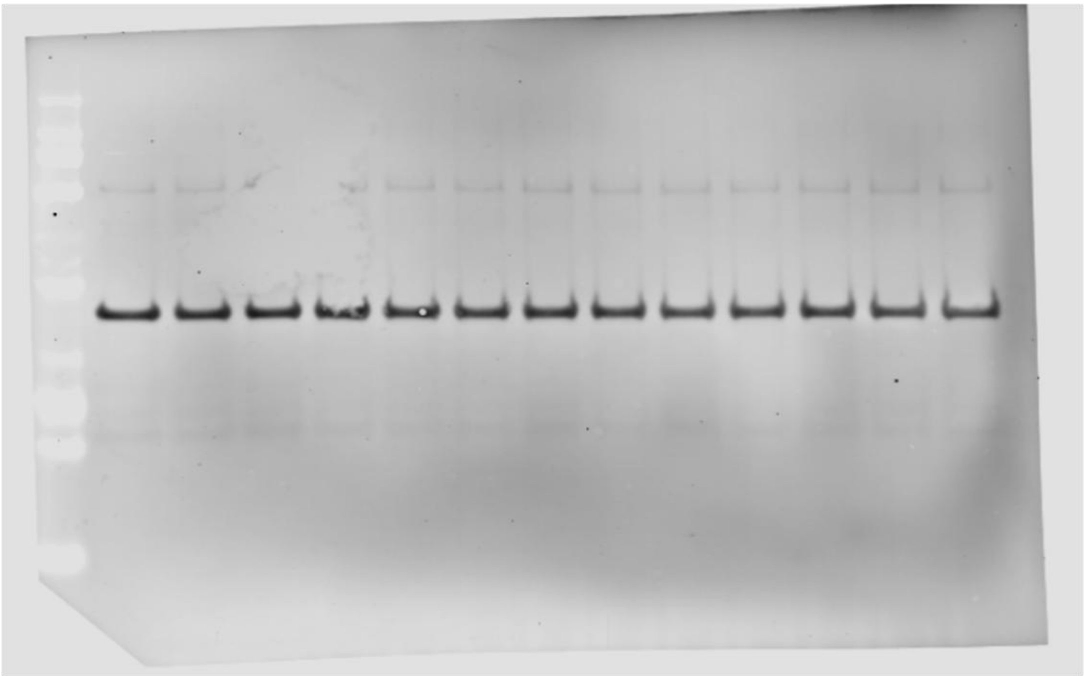

Fig. 3e

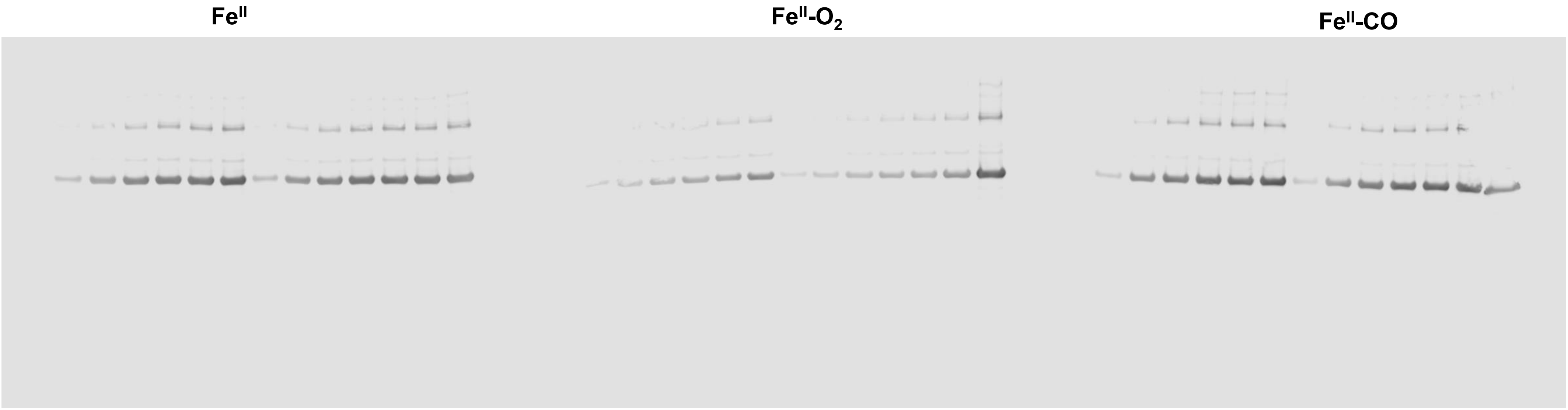

Fig. 3f

Fe^{II} | Fe^{II}-O_2 | Fe^{II}-CO

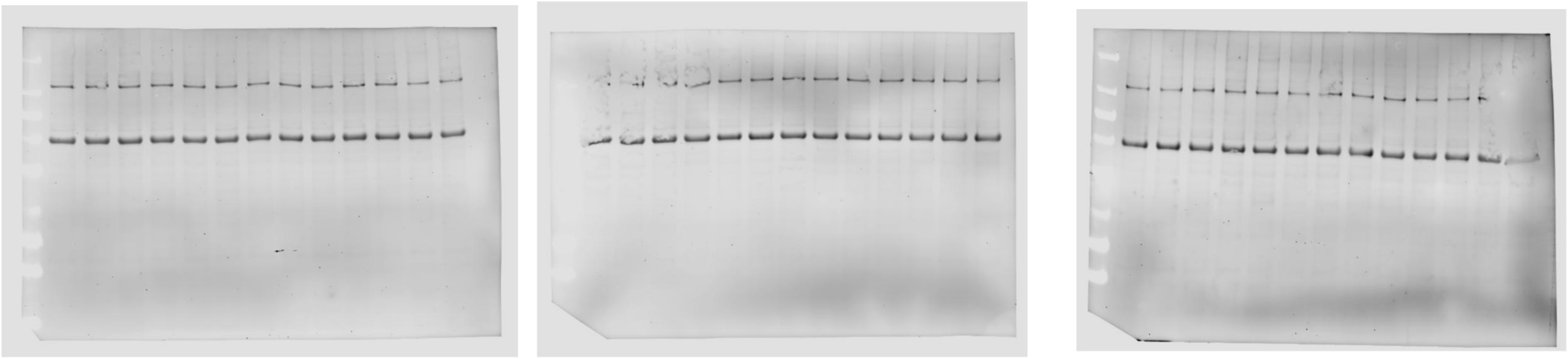

SI Fig. 6a

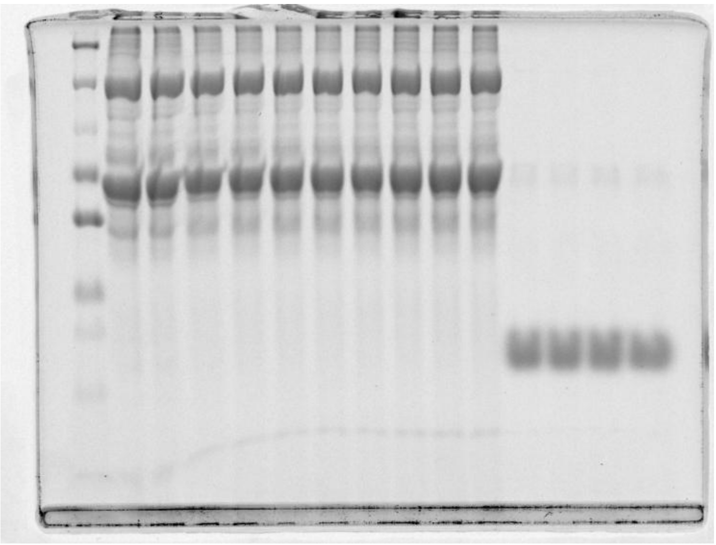

SI Fig. 6b

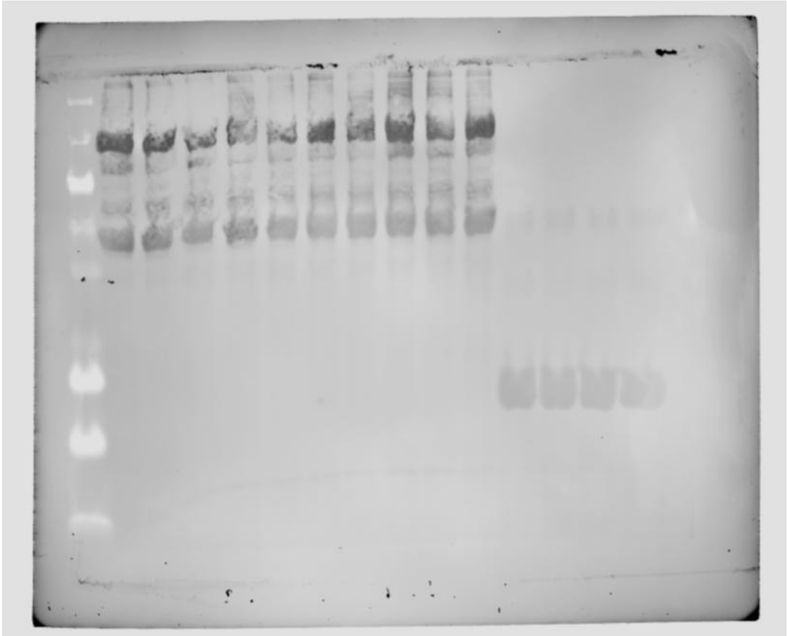

SI Fig. 11a

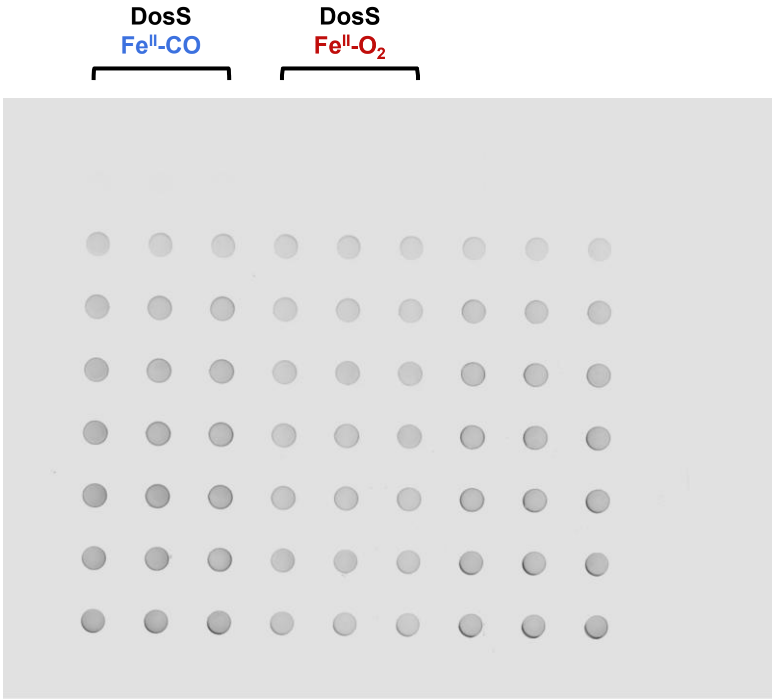

SI Fig. 11b

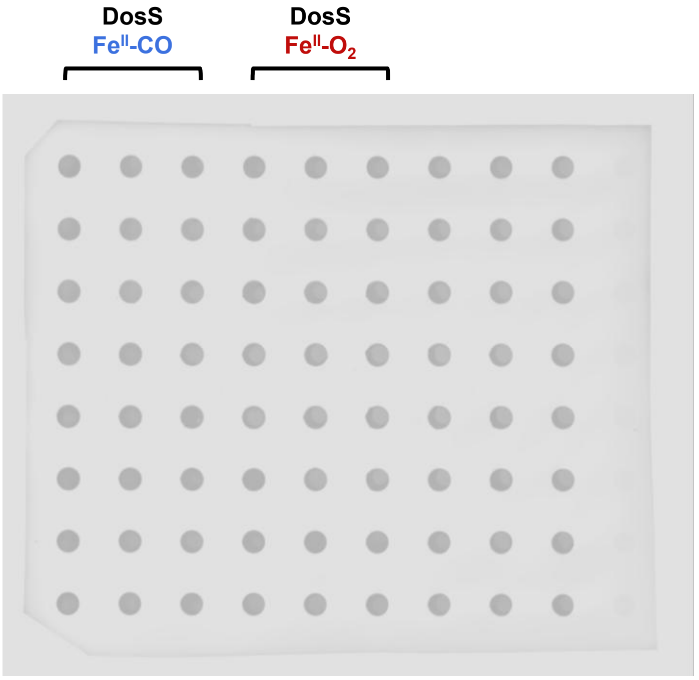

SI Fig. 12

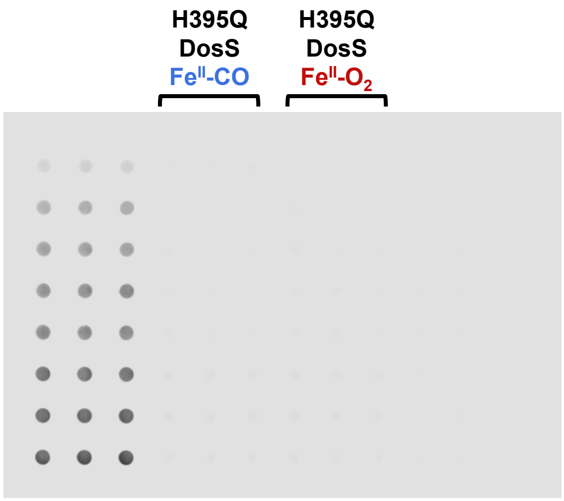

Supplement: Supplementary file 2 — Supplementary Material 2 [file 775_2026_2145_MOESM2_ESM.pdf]
